# Informed consent and community engagement in open field research: lessons for gene drive science

**DOI:** 10.1186/s12910-019-0389-3

**Published:** 2019-07-27

**Authors:** Jerome Amir Singh

**Affiliations:** 1 Centre for the AIDS Programme of Research in South Africa (CAPRISA), Nelson R. Mandela School of Medicine, University of KwaZulu-Natal, Durban, South Africa; 2 Dalla Lana School of Public Health, University of Toronto, Toronto, Canada

**Keywords:** Informed consent, Vector borne diseases, Gene drive, Genetically modified mosquitos, Malaria, Novel technology, Community engagement, Stakeholder engagement

## Abstract

**Background:**

The development of the CRISPR/Cas9 gene editing system has generated new possibilities for the use of gene drive constructs to reduce or suppress mosquito populations to levels that do not support disease transmission. Despite this prospect, social resistance to genetically modified organisms remains high. Gene drive open field research thus raises important questions regarding what is owed to those who may not consent to such research, or those could be affected by the proposed research, but whose consent is not solicited. The precise circumstances under which informed consent must be obtained, and from whom, requires careful consideration. Furthermore, appropriate engagement processes should be central to any introduction of genetically modified mosquitos in proposed target settings.

**Discussion:**

In this work, international guidance documents on informed consent and engagement are reviewed and applied to the genetically modified mosquito research context. Five analogous research endeavours that involve area-wide / open field experiments are reviewed. The approach of each in respect to the solicitation of individual informed consent and community engagement are highlighted.

**Conclusions:**

While the solicitation of individual informed consent in host settings of gene drive field trials may not be possible or feasible in some instances, local community and stakeholder engagement will be key to building trust towards the proposed conduct of such research. In this regard, the approaches taken by investigators and sponsors of political science field research and weather modification field research should be avoided. Rather, proponents of gene drive field research should look to the *Eliminate Dengue* field trials, cluster randomised trials, and pragmatic clinical trials for guidance regarding how the solicitation of individual informed consent of host communities ought to be managed, and how these communities ought to be engaged.

## Background

Vectors are living organisms that can transmit infectious diseases between humans or from animals to humans [1]. The role of vectors in diseases transmission is a relatively recent discovery and emerged through a series of advances in clinical practice in the seventeenth century. Changes in the practice of medicine in the 1600s centring on careful clinical observation, differentiation, and specific diagnosis, led to the search for specific, as opposed to general, causes of illness, and resulted in the differentiation of diseases [2]. In 1877, the discovery that mosquitoes transmitted filariasis from human to human led to the disease transmission role of vectors being uncovered in relation to malaria (1898), yellow fever (1900), and dengue (1903) [2, 3]. By 1910, the role of vectors was uncovered in the transmission of a range of other diseases, including African sleeping sickness, plague, Chagas disease and sandfly fever [2, 3].

Four vector-borne diseases – dengue, malaria, plague, and yellow fever – have collectively accounted for the majority of human morbidity and mortality that occurred between the 17th and 20th centuries [4, 5]. The development of strategies aimed at suppressing the population of mosquitoes responsible for transmission of dengue, malaria and yellow fever temporarily reduced the rate of human morbidity resulting from these diseases [5, 6]. Nevertheless, there has been a resurgence of several vector-borne diseases that were otherwise generally containable, “in new geographic locations” in recent times. Concurrently, a number of previously unknown pathogens and vectors have “triggered disease outbreaks in humans” [5, 6]. Today, vector-borne diseases threaten the majority of the world’s population, is attributable to more than 17% of all human infectious diseases, and claims more than 700,000 fatalities annually [1].

Malaria is the world’s deadliest vector-borne disease, accounting for an estimated 445,000 deaths globally in 2016, with 91% of those fatalities occurring in Africa [7]. The development of the CRISPR/Cas9 gene editing system has generated new possibilities for the use of gene drive constructs to reduce or suppress mosquito populations to levels that do not support disease transmission [8–10]. More specifically, the deployment of gene drive mosquitoes may offer a potential biocontrol tool for the elimination of malaria in Sub-Saharan Africa [11]. Despite this prospect, social resistance to genetically modified organisms remains high in some settings. In 2016, a proposed field trial of genetically modified *Aedes aegypti* mosquitoes to prevent Zika virus transmission in Florida generated major controversy, with residents signing petitions and even erecting “no consent” signs on their lawns in opposition to the proposed trial [12]. This underscores that the introduction of genetically modified mosquitos (GMM) could face challenges in other target settings, notwithstanding the potential beneficial impact of GMM on human health in those settings. The World Health Organisation (WHO) has noted that “some commentators have argued that informed consent will be necessary to ensure that GMM trials are conducted ethically. However, the precise circumstances under which informed consent must be obtained, and from whom, requires careful consideration” [13]. This work reviews five analogous research endeavours that involve area-wide / open field experiments. The approach of each in respect to the solicitation of individual informed consent and community engagement may hold lessons for GMM field trials.

## Discussion

Before different models of informed consent and community engagement can be explored, it is important to review the position of major research ethics guidance documents on informed consent and community engagement.

### Global research ethics guidance on informed consent and engagement

The *Declaration of Helsinki* (hereinafter *DOH)* [14], published by the World Medical Association, along with the *International Ethical Guidelines for Health-Related Research Involving Humans* [15] (hereinafter *CIOMS Guidelines*), published by the Council for International Organizations of Medical Sciences (CIOMS) – an international, non-governmental, non-profit organization established jointly by the WHO and the United Nations Educational, Scientific and Cultural Organisation (UNESCO) – are universally regarded as the world’s leading international research ethics guidance documents. Both instruments provide guidance on informed consent. However, it may be argued that the *DOH* is not applicable to research focused on gene drive constructs to reduce or alter vector populations as the *DOH* is “addressed primarily to physicians” and its guidance is restricted to “medical research” [14]. In its 2016 iteration, the *CIOMS Guidelines* positioned itself wider – from “biomedical research” to “health-related research involving humans” *–* and does not restrict its guidance to primarily physicians. The *CIOMS Guidelines* provides instructive guidance on community engagement and informed consent, which are essential factors to consider in the conduct of field research.

Guideline 7 of the 2016 version of the *CIOMS Guidelines* governs community engagement and states:

“Researchers, sponsors, health authorities and relevant institutions should engage potential participants and communities in a meaningful participatory process that involves them in an early and sustained manner in the design, development, implementation, design of the informed consent process and monitoring of research, and in the dissemination of its results.”

Guideline 10 of the 2016 iteration of the *CIOMS Guidelines* governs informed consent and states:

Researchers must not initiate research involving humans without obtaining each participant’s individual informed consent or that of a legally authorized representative, unless researchers have received explicit approval to do so from a research ethics committee …. A research ethics committee may approve a modification or waiver of informed consent to research if: (i) the research would not be feasible or practicable to carry out without the waiver or modification; (ii) the research has important social value; and (iii) the research poses no more than minimal risks to participants. Additional provisions may apply when waivers or modifications of informed consent are approved in specific research contexts.

With the *DOH* characterising itself as a “statement of ethical principles for *medical research* involving human subjects” and “addressed primarily to physicians”, and the focus of the *CIOMS Guidelines* primarily centred on the ethical conduct of *clinical trials,* neither of these guidance documents are apt for GMM field research, which is multi-disciplinary in nature and involves a different testing and development pathway to clinical trials. Accordingly, in 2014, the WHO published a *guidance framework for testing of genetically modified mosquitoes* (hereinafter *Guidance*) [13]. The WHO *Guidance* highlights 3 levels in the authorisation process pursuant to GMM field trials: (i) informed consent at the individual level, where relevant; (ii) community authorisation; and (iii) regulatory / government permission. The *Guidance* defines informed consent as “the process intended to ensure that human subjects who will be observed or involved in a research activity are fully and explicitly advised of all risks, costs or inconveniences they may bear as a result of participating as a research subject, and voluntarily agree to accept or bear those risks and costs.”

The *Guidance* endorses McRae et al’s four proposed criteria [16] “to determine whether an individual is a research participant, and therefore should normally give informed consent as a condition of their participation: (1) If an individual is directly intervened upon by an investigator; (2) If an individual is deliberately intervened upon via manipulation of the individual’s environment by an investigator; (3) If an individual interacts with an investigator for the purpose of collecting data; or (4) if an investigator obtains identifiable private information about the individual for the purpose of collecting data” [16]. Based on these criteria the *Guidance* concluded that “caged field trials or open releases of GMMs in the context of a research trial would not satisfy the requirements of the first two criteria, since no individual is intervened upon directly or deliberately, even if they live in close proximity to the cages or release sites” [13]. The *Guidance* notes that “the third and fourth criteria focus on the interactions between investigators and individuals who play some special role in generating or facilitating the collection of study data” [13]. Accordingly, the *Guidance* concluded that “simply living in the vicinity of a GMM release is not sufficient grounds to require informed consent from any individual for an open release of mosquitoes” [13]. However, “the interactions with individuals and households for the purposes of data collection in trials with both entomological and epidemiological endpoints are likely to give rise to individual, or household---level identifiable data and, therefore, in the absence of specific exceptions or waivers, will require informed consent” [13].

According to the *Guidance*:

"In GMM trials there is a wide range of interactions with the host community, but only a select few that are associated with data collection. In early phase trials, this would pertain to individuals who agree to complete surveys or participate in interviews for research purposes associated with the GMM trial [13]. It would also pertain to those homeowners who agree to the placement of mosquito traps for monitoring purposes, or who permit researchers access to their homes for the purpose of collecting mosquitoes [13]. In particular, mosquito collection in homes for research purposes is likely to be linked to global positioning system (GPS) data, which would be required for spatial analyses of the spread and species composition of mosquitoes after releases [13].

When these GPS data are highly precise, they will effectively tie the associated mosquito data to specific households, thus rendering the data identifiable at this level even if they are not personal in nature [13].

Since it is the household that is identified, and not an individual, the consent of the head of the household or her/his designate is more appropriate than a requirement for all members of the household to provide informed consent [13].

And given the extremely low levels of risk associated with these types of data collection activities, institutional review boards might further consider modifications of normal consent procedures, such as verbal consent or full waivers of informed consent, as long as all other necessary permissions and protections have been secured [13]. As trials progress from primarily entomological endpoint designs to incorporate epidemiological endpoints, such as incidence of new infections with dengue or malaria, they will require the collection of blood and other forms of clinical data [13]. In both cases, the data collected will constitute identifiable personal information and individual informed consent will be required" [13].

The reliance on the solicitation of authorisation / consent from only the head of the household on behalf of all household inhabitants has its roots in the Roman notion of the *paterfamilias* (father / head of a Roman family). Historically, the *paterfamilias* was the oldest living male in a household and exercised autocratic authority over his extended family. This notion was reinforced in the *Defensor pacis,* the seminal work of Marsilius of Padua, a fourteenth century Italian scholar, and political scholar trained in medicine. The *defensor pacis* asserts the notion that the discretionary authority of the head of the household is complete and unassailable, and that the realm of the household is governed according to the discretion of its head [17]. In today’s world, the head of the household is not restricted to senior males. The solicitation of consent from the household head for research purposes is endorsed by the WHO in other research contexts too [18]. Household head authorisation / consent has furthermore been recognised and utilised by local researchers working in some rural African settings [18], and by local researchers working with illiterate populations in some settings too [19, 20].

In relation to ethics and public engagement with the host community, guidance point 31 of the *Guidance* states:

"Researchers have ethical responsibilities to people living within a trial site. For that subset of individuals classified as “human research subjects” according to standard regulatory criteria, informed consent obligations will apply. However, there may be many individuals living within a trial site who are not, in a traditional sense, subjects of the research at hand, but who nonetheless may be affected by the conduct of research.

Community Engagement addresses ethical obligations to these people, including undertaking procedures that would be expected to identify them, advising them that they may have interests at stake, finding out what concerns they may have, responding to those concerns, and reaching some form of agreement about whether the trial should proceed."

Guidance point 43 also states: “Informed public involvement and consent in the GMM regulatory decision process is a necessity if implementation is to occur without adverse public reaction. Regulatory processes often include formal public consultation opportunities.”

In its summary on ethics and public engagement, the *Guidance* states:

"Beginning in Phase 2 and expanding in Phase 3, community engagement activities are intended to address ethical responsibilities beyond the formal permissions required at the individual level (informed consent) and the governmental level (regulatory compliance).

The concept of “community authorization” entails providing those living in the trial site with methods to give or withhold agreement for trial activities, and to identify elements that they believe to be important for the research to continue.

During field testing, scientists also should expect to interact with third parties who express interest in the activity and its outcomes, both to ensure that the project is well understood and to avail the project team of information and insights that such interested parties might provide. However, given the diverse range and varied degrees of interest of third parties, there is not the same level of obligation to seek them out proactively to ensure that they are informed about the project, as is the case with those directly affected."

While research ethics guidance documents and the WHO *Guidance* recommend that engagement with communities, publics, and stakeholders should be central to proposed research activities, it is important to reflect on who should drive or steer this process and why building trust is an important goal worth pursuing. These important factors, including in relation to gene drive research, have been explored elsewhere [21–23].

### Stewardship and trust

While researchers and sponsors traditionally drive engagement activities, in light of the complicated matters concerning community engagement and ethics that are inextricably linked with gene drive technology [5], and because researchers and sponsors may be conflicted in driving the engagement process, the ‘Scientific Working Group considering pathways to deployment of gene drive mosquitoes as a potential biocontrol tool for elimination of malaria in Sub-Saharan Africa’ has recommended that “an ethics advisory group comprising experts external to the project would be an important mechanism to supplement the input from community advisory boards or other community engagement activities, providing additional and broader perspectives” [5, 24].

Even notwithstanding the involvement of an external ethics advisory group in engagement initiatives, it is important to stress that engagement should never be undertaken simply because it is a means to an end. Researchers have an ethical obligation to advance trustworthy gene drive science. Moreover, engagement should never be regarded as simply a series of minimum actions or steps to be taken to facilitate the conduct of gene drive science. Doing so may amount to procedural justness, but does not count as substantive justness. Rather, the establishment of trust between researchers and sponsors, on the one hand, and stakeholders, publics, and communities on the other hand, is morally important and worth seeking as an end-in-itself. It has been argued that “characteristics or virtues such as fairness, openness, transparency, consistency, and also dedication to ethics and ethical research, can be seen as indications of an institutions’ moral character and promote trustworthiness” [25, 26]. The US Institute of Medicine has further argued that institutions have an ethical duty to create an environment “that promotes responsible conduct by embracing standards of excellence, trustworthiness, and lawfulness that inform institutional practices” [25, 27]. Engagement should be seen as key to developing an environment conducive to fostering “responsible conduct” [25]. In its report on gene drive research, the US National Academy of Sciences, Engineering and Medicine (NASEM) stressed that engagement is crucial to the oversight of gene drive research for several reasons [28].

First, communities and stakeholders have knowledge that is essential to understanding the complex and variable social, political, economic, and ecological contexts in which gene drives will operate. Second, principles of justice demand both transparency in and well-informed consent to any future (experimental) trials that may affect communities of people and landscapes. The inability to maintain transparency with respect to data can exacerbate apprehension and distrust by forming the notion that scientists are capable of willingly withholding significant information that should otherwise be available and accessible [5]. Third, engagement creates opportunities for mutual learning that foster forward thinking, reflective deliberation, and the building of trust among diverse groups. It has further been argued that engagement satisfies four ethical goals—enhancing protection, enhancing benefits, creating legitimacy, and sharing responsibility [29].

These cumulative factors underscore why researchers ought to perform engagement activities that build trust. As the informed consent process is regarded as central to establishing trust, the *Guidance*’*s* position that the solicitation of informed consent may not be necessary in the conduct of certain aspects of GMM field research and its nod to “community authorisation” processes raise questions about whether there is precedent for such practices [13]. To this end, several scientific disciplines engage in research activities that do not involve the solicitation of individual informed consent. Some engage in community engagement activities pursuant to non-consensual research activities, while others do not. These approaches will be highlighted for two reasons: (i) to demonstrate precedent for such approaches in the science context; and (ii) to enable a normative ethics assessment of each approach in respect of their appropriateness to gene drive science.

### Population-based research

Population-based research may be described as “human subject research where the objectives aim to improve the health of populations and discover interventions that raise the baseline health status of entire communities” [30]. While adults may never be enrolled in clinical research in absence of their voluntary consent, in population-based research, competent adults may be exposed to an experimental public health intervention without their permission.

#### The eliminate dengue initiative: Wolbachia-based open field trials

*Wolbachia pipientis* is a maternally inherited intracellular bacterium that is found in a wide range of arthropod species and filarial nematodes, with approximately 40% of insect species infected [31]. Wolbachia infections have been found to confer protection for their insect hosts against a range of pathogens including bacteria, viruses, nematodes, and the malaria parasite. Despite the existence of numerous insects that are naturally infected by Wolbachia, the bacteria is incapable of being transmitted to homeothermic species of animals, specifically mammals and birds. Thus, humans cannot be infected with Wolbachia [32].

Wolbachia is not usually found in the *Aedes aegypti* mosquito, the primary species responsible for transmitting human viruses such as Zika, dengue and chikungunya [32]. When research demonstrated that Wolbachia introduced into mosquitoes interfere with pathogen transmission and influence key life history traits, such as lifespan [33], and could potentially be deployed as a strategy to suppress dengue [34, 35], *Eliminate Dengue*, an international research program focusing on open field releases of Wolbachia-infected mosquitos, was developed to control dengue transmission. While the involvement of human volunteers for mosquito “blood-feeding” in pursuance of such trials requires the approval of a research ethics committee and written informed consent from the volunteers [36], the approaches taken in regard to solicitation of informed consent in Australian, Vietnamese, and Indonesian open field trials bear noting as they could be instructive to proposed open field gene drive malaria research. At least three approaches to the solicitation of informed consent has emerged from this research programme.

##### Australia: prior community engagement; individual informed consent sought from one member of consenting households in host community

In Cairns, Australia, community engagement activities preceded Wolbachia-based field trials [37]. The research team worked on a community engagement strategy a year prior to the actual community engagement activities, which ran over 2 years preceding the release [36]. Researchers used a mixed method social research approach and engagement to determine how the communities of Cairns (particularly planned release locations) would like to be engaged; what constitutes authorization/consent; and “acceptance and non-acceptance” as well as issues surrounding “acceptability and non-acceptability” [36]. During the community engagement activities, researchers taught the communities about Dengue and the acceptability of Wolbachia utilizing a lay-knowledge approach [36]. Researchers employed focus groups, in-depth interviews, participant observations and questionnaires to gauge the full range of participant views [37]. Thereafter, researchers confirmed the views of residents by way of telephone surveys; representative population samples; and random sampling [36]. Consequently, researchers found that the participants were receptive to the proposed release but had certain misgivings. The participants wanted assurance that Wolbachia would not detrimentally affect human health and the environment. In order to quell the concerns of residents, the research team initially composed a compendium of the most compelling literature in the field and incorporated this information into communication and engagement materials [35]. Thereafter, researchers undertook experimental evaluations, conducted available observations, and reviewed the knowledge of the approach of the literature to ensure that Wolbachia would not jeopardize the health of human, animals and the environment [35].

Once community engagement activities concluded, researchers requested written consent from the residents of all households in the selected release locations [30]. Each household had to provide consent for three specific activities: (a) Pre-release suppression (water extraction from potential breeding sites); (b) release of wolbachia-infected mosquitos; and (c) installation of monitoring traps [35]. 97% of residents in host areas provided consent to participate whilst 3% did not [35]. During the time leading up to the planned release, researchers were involved in on-going engagement activities, including household surveys, monthly meetings, and had free contact numbers [35]. Mosquito control was offered to households that expressed concern [35]. Residents were given periodic updates via publicly accessible forms of media (local newspaper, local radio stations, and paid advertisements) [35]. One to 4 weeks prior to the open field releases, the researchers visited every consenting household but were only able to access approximately half of the households [35]. Subsequently, release commenced on January 2011 for 10 weeks and was rotated between the two sites. Monitoring occurred every fortnight and traps were 7 days post deployment [35]. Consent was obtained from one member of every fourth house in affected areas where residents had agreed to participate in the study, for the release of 50 or so insects [38].

When consent to release the mosquitoes was denied by a household, researchers ensured that the household’s neighbours in adjacent properties were not subjected to the release of the mosquitoes, and they were offered installed traps on their properties [24, 37]. It was noted that whilst “residents appreciated these gestures, tensions occasionally arose between the (research team’s) commitment not to foist the technology on the community, and the wishes of individuals who refused to permit releases on their property” [24, 37].

##### Jogyakarta, Indonesia: prior community engagement; release of Wolbachia-infected mosquitos done in public areas near homes, or, with prior consent, on resident’s properties

In Jogyakarta, Indonesia, upon securing community support and regulatory approval, adult mosquitoes were released in public areas located close to residential homes, and if permission was provided by residents to release the mosquitoes on their properties, researchers proceeded to do so by placing containers on the properties [32, 39]. Each container not only held the mosquitoes, but contained eggs infected by the Wolbachia bacteria, which would eventually hatch and release Wolbachia-infected mosquitoes [32, 39]. Monitoring traps that were introduced prior to the release of the mosquitoes were utilized for the duration of the release, as well as after the release [32, 39]. The traps were installed in and around properties and was used to frequently gather samples “of mosquitoes from the field trial areas” for analysis in the lab [32, 39].

##### Tri Nguyen Island, Vietnam: prior community engagement; consent solicited from every household in field trial area

In Tri Nguyen Island, Vietnam, a representative from every household on the island was asked to provide their consent for a release of Wolbachia-infected mosquitos. Of these, more than 95% agreed to support the release [40]. Researchers focused primarily on residents, but also engaged with health providers, government officials, and scientists with responsibilities at local, national and regional levels. In order to devise an appropriate Wolbachia strategy, researcher also employed a mixed method social research approach to inform residents and gauge their concerns using in-depth interviews, questionnaires, household surveys, and purposeful sampling. Researchers also provided training to scientists and entomologists, and liaised with government leaders and health providers.

**Table:** 

**Commentary and recommendation** Wolbachia-based open field trials offer an analogous example of how the introduction of an experimental intervention in a field-trial context may or may not necessitate the solicitation of individual informed consent. In Australia and Vietnam, investigators deemed the solicitation of informed consent from at least one member of a household a prerequisite for the release of Wolbachia-infected mosquitos on that household’s property. This approach is endorsed by the WHO *Guidance*. In Indonesia, wolbachia-infected insects were released in public areas near households without the consent of nearby households, or, with the consent of a household member, wolbachia-infected mosquitos were released on that household’s property. Such strategies may be instructive for the conduct of gene drive field trials, which also involves the introduction of an experimental intervention in a field trial context. However, given that gene drive research involves the introduction of GM mosquitoes, which may raise more concerns than the Wolbachia strategy, investigators should devise a bespoke community and stakeholder engagement process, and consider conducting relevant preceding social science research, to gauge perspectives of the host community on the technology. If the perspectives of the community are meaningfully addressed, such an approach could facilitate the realisation of the four ethical goals of engagement, namely, enhancing protection, enhancing benefits, creating legitimacy, and sharing responsibility.	

#### Cluster randomised trials

Cluster randomised trials (CRTs) offer an example of a population-based area-wide introduction of an intervention that may or may not require the solicitation of individual informed consent. A cluster randomised controlled trial is a type of randomised controlled trial in which groups of subjects (as opposed to individual subjects) are randomised to an intervention group or control group. In such studies, some clusters receive the experimental intervention, and other clusters (the control groups), do not. All ‘participants’ in a cluster are offered only the intervention or its assigned alternative. Some CRTs offer participants the opportunity to ‘opt out’ of participation in the trial. CRTs may be distinguished between “individual-cluster” trials and “cluster-cluster” trials. In individual-cluster trials, a study intervention -- such as a vaccination – is directed at individual cluster members. Due to the fact that treatment is administered to individuals, it is possible for individuals to provide consent for the treatment “offered within their cluster” [41]. Accordingly, informed consent is often obtained in individual-cluster trials. The Thibela TB trial is an example of a large population-based cluster randomised trial (> 78,000 participants) where individual informed consent was solicited from participants prior to randomisation [42]. In this study, entire mining communities were randomised to receive either the experimental intervention (isoniazid as *preventive* therapy) or the existing standard of care. The investigators solicited individual informed consent from participants and undertook intensive community engagement activities in furtherance of the trial [43]. These engagement initiatives may be instructive to proposed gene drive field trials, even if individual informed consent is not to be solicited from communities hosting such trials.

During “cluster-cluster” trials, it can be virtually impossible to solicit informed consent from each individual within the cluster, and because the study “is delivered at the cluster level” it could be extremely difficult for individuals within the cluster “to avoid the intervention if they do not wish to participate in the CRT” [41]. Consequently, “individuals cannot act independently” and “the autonomy principle is lost” [41, 44]. Moreover, when faced with large clusters it may be impossible to solicit informed consent from each member from a logistical perspective [41]. Accordingly, obtaining informed consent in the context of “cluster-cluster” trials “may not be meaningful or feasible” [41].

A community-randomized controlled trial conducted in Pakistan to determine the efficacy of indoor residual spraying with alphacypermethrin, to prevent falciparum and vivax malaria, offers an example of a cluster-cluster trial where investigators deemed the solicitation of individual informed consent to be unfeasible [45]. In Pakistan, malaria is a major disease [41]. Thus, the use of indoor insecticides is the most prevalent preventative measure in households [41]. The aim of this particular CRT was to “test the effectiveness of a new insecticide –alphacypermethrin – in controlling malaria rates in rural Pakistan” [41]. The study was conducted over an area of 180 km^2^ in Punjab province. The study area “was divided into nine sectors and each was randomized to spraying with one of two preparations of the insecticide or a no spraying control” [41]. “In the two intervention arms of the study, all living quarters, storage rooms, and animal quarters were sprayed once with the insecticide” [41]. “Survey teams visited 400 houses in each district every two weeks to identify new cases of malaria by symptom report and, when indicated, a blood smear to look microscopically for the parasite” [41]. “Additionally, a cross-sectional survey collected blood smears from 200 to 300 school children in each sector before and after the intervention period” [41]. Village elders were informed of the study and gave their permission for the study to be conducted" [41].

All residents within the study area were recipients of deliberate intervention as a result of the “manipulation of their environment and, hence qualified as human research subjects” [41]. Still, obtaining individual consent from all of the study subjects would have been extremely challenging since the study consisted of intervention activities (“spraying all living quarters, storage rooms, and animal quarters within a geographic area”) would have been virtually impossible for subjects to avoid [41]. Consequently, the refusal of consent would have been futile [41]. Further, given that each study sector, of which there were nine, comprised of “approximately 2,000 people living in 400 homes” soliciting informed consent from residents, “would have rendered the study infeasible” [41].

**Table:** 

**Commentary and recommendation** Cluster-cluster RCTs offer an analogous example of how the introduction of an experimental intervention in an open field-trial context may not necessitate the solicitation of individual informed consent, and where the permission of village elders for the conduct of the study, may be an acceptable surrogate for individual informed consent to conduct a field trial. Such a strategy may be instructive for the conduct of gene drive field trials, although an appropriate community and stakeholder engagement process should precede and accompany the conduct of a gene drive field trial. The Thibela TB trial offers a model of how such engagement can be realised. Such an approach could help ensure the incorporation of a community’s views and its participation in research, and accordingly, satisfy the four ethical goals of engagement, namely, enhancing protection, enhancing benefits, creating legitimacy, and sharing responsibility.	

#### Pragmatic clinical trials

Pragmatic Clinical Trials (PCTs) typically take place in clinical-care settings and often compare existing and/or approved interventions or therapies, any of which may constitute standard care for a given condition [35]. Pragmatic clinical trials have been described as “designed to inform decision makers about the benefits, burdens, and risks of health interventions in real-world settings” and often using, “for research purposes, data collected in the course of clinical practice” [46]. Research participants in pragmatic clinical trials may be characterised as follows [46, 47]:

- *"Direct participants:* (a) individuals being directly intervened upon and/or (b) individuals from whom personal identifiable data are being collected for the purposes of the pragmatic clinical trial [46].
- *Indirect participants*: individuals who are (a) not identified as direct participants and (b) whose rights and welfare may be affected by the intervention through their routine exposure to the environment in which the intervention is being deployed [46].
- *Collateral participants:* patient groups and other stakeholder communities who may be otherwise affected by the occurrence and findings of the pragmatic clinical trial" [46].

The US Common Rule defines a “human research subject” as “a living individual about whom an investigator (whether professional or student) conducting research obtains (1) data through intervention or interaction with the individual, or (2) identifiable private information” [46].

The Common Rule defines “interaction” as including “both physical procedures by which data are gathered (for example, venipuncture) and *manipulations of the subject or the subject’s environment that are performed for research purposes*” (italicised for emphasis) [46].

In PCTs, informed consent is generally solicited from direct participants, although ethics committees may be approached to waive the solicitation of consent from such participants. However, *informed consent is not solicited for indirect or collateral participants, despite the possibility of these individuals being affected because of manipulation of the subject’s environment for research purposes.* Seen in this context, indirect and collateral participants in PCTs are analogous to potentially affected parties in gene drive field trials. Community engagement strategies have been proposed for PCTs [49], which may be instructive to the planning of gene drive field trials.

**Table:** 

**Commentary and recommendation** Community engagement strategies proposed for PCTs should be considered in the preparation and conduct of gene drive field trials. Such an approach would ensure the incorporation of a community’s views and its participation in research, and accordingly, satisfy the four ethical goals of engagement, namely, enhancing protection, enhancing benefits, creating legitimacy, and sharing responsibility. However, PCTs offer precedent for instances where indirect or collateral participants in research does not necessitate the solicitation of informed consent from such parties. This is analogous to individuals who may be indirectly or collaterally affected through the conduct of gene drive field trials. According to the WHO *Guidance,* even if individuals live in close proximity to the caged trials or release sites, it is ethically permissible to not solicit informed consent from such individuals as they are not being intervened upon directly or deliberately.	

#### Political science field experiments

“Field experiments are manipulations conducted in the real world rather than in a laboratory” [48]. In political science field experiments, the researcher’s intervention takes place in an environment where the researcher has only limited control beyond the intervention conducted and the relationship between the researcher and the subject is conducted often through variables outside of the researcher’s control.

Generally, field experiments consist of treatments or interventions that are administered without the solicitation of informed consent from study subjects [48].

Non-consensual political science experiments have been conducted since at least the mid-1920s. The first known political science experiment was on voter turnout in Chicago. In this experiment, the investigator randomly assigned districts to receive information on voter registration and encouragements to vote [50]. Informational field experiments (IFE) involve sending subjects’ information, then observing their behaviour. Such experiments have been described as “usually election-related studies” [48]. Typically, subjects are provided information relating to voting activities or candidates up for election. Thereafter, researchers will observe whether subjects did vote, or more specifically, which candidate was voted for [48]. One of the main aspects incorporated into the design of political science experiments is that subjects are generally unaware that they are, in fact, subjects in a study [48]. Soliciting informed consent is usually omitted from the design of the study, which “often employ deception as investigators may purport to be from a non-existent interest group or contain (provide) other misleading information” [48].

Whereas the *individual risk of harm* in most IFEs is small, the *aggregate effects* are potentially large. These aggregate risks include affecting other citizens in a political system. Although subjects are not subjected to any individual harm when they accept election flyers, they may experience collective harm depending on the outcome of an election [48]. For example, the election of a conservative candidate may result in liberty or benefit restrictions, which may ultimately detrimentally impact upon the subject of the research. Harm may also extend directly to non-subjects. For example, political science experiments will inevitably result in the benefit or harm of either a constituency or candidate depending on the outcome of the votes [48].

In political science field experiments, no community engagement precedes the conduct of the research. Instead, waiver of informed consent is sought from governing research ethics committees, or in some instances, no approval is sought from governing ethics committees. This was the case in a controversial political science experiment conducted in Montana in 2014, which elicited outrage in the US and garnered prominent media coverage [51, 52].

**Table:** 

**Commentary and recommendation** In political science field experiments, no community engagement precedes the conduct of the research. Instead, waiver of informed consent is sought from governing research ethics committees, or in some instances, no approval is sought from governing ethics committees. Such an approach is not recommended for gene drive field trials as it does not ensure the incorporation of a community’s views and its participation in research, and accordingly, fails to satisfy the four ethical goals of engagement, namely, enhancing protection, enhancing benefits, creating legitimacy, and sharing responsibility. Instead, appropriate community and stakeholder engagement, and relevant social science research should precede gene drive field research.	

#### Geoengineering and weather modification experiments: “cloud-seeding” as an intervention for water resource management and weather hazard mitigation

Weather modification is the intentional human activity of manipulating or altering the weather and falls under the wider field of geo-engineering or environmental modification. The most common form of weather modification is the “seeding” of clouds with materials such as silver iodide crystals to increase rain or snow, usually for the purpose of increasing the local water supply or weather hazard mitigation. Weather modification experiments have been conducted in the US since at least the 1940s [53]. The US state of Montana is an example of a setting that currently engages in weather modification experimentation and which has enacted regulations to govern weather modification [54] and research thereon [55]. Such is the alarm that environmental modification triggers, that in 1976, the United Nations General Assembly passed the Convention on the Prohibition of Military or Any Other Hostile Use of Environmental Modification Techniques. The Convention prohibits the military or other hostile use of environmental modification techniques having widespread, long-lasting or severe effects. It opened for signature on 18 May 1977 in Geneva and entered into force on 5 October 1977. In 2013, at least 42 countries globally were known to be engaging in weather modification activities / experimentation [56]. A defining feature of weather modification experimentation is that no prospective informed consent is sought from persons in areas that could be affected by the experiments. Moreover, no community engagement has typically preceded such experimentation. As a result, geo-engineering / weather modification science is treated with suspicion. Because such science: (a) is undertaken without prior public consultation or engagement, (b) is conducted non-consensually, and (c) may have inadvertent consequences (for example, cloud-seeding to mitigate violent hail storms may reduce rainfall, thus affecting agricultural farming) [57], some sectors of civil society describe weather modification science and climate engineering as “the greatest assault of all against life on Earth” [58].

**Table:** 

**Commentary and recommendation** Weather modification field experimentation is not a good model for gene drive field trial research as it does not ensure the incorporation of a community’s views and its participation in research, and accordingly, fails to satisfy the four ethical goals of engagement, namely, enhancing protection, enhancing benefits, creating legitimacy, and sharing responsibility. Even if the solicitation of individual informed consent is not deemed feasible in the context of gene drive field research, stakeholder engagement will be key to preventing gene drive science from being treated with the high levels of mistrust that currently characterise weather modification science.	

## Conclusions

Gene drive field research is a nascent science. The potential for fear and mistrust is high. While the *CIOMS Guidelines* may arguably not apply to vector field trials which do not directly involve human participants, it posits potential factors that may be forwarded to justify the waiver of the solicitation of individual informed consent in host settings. These include that (i) the research would not be feasible or practicable to carry out without the waiver or modification; (ii) the research has important social value; and (iii) the research poses no more than minimal risks to participants (which is the case in phase 2 field trials). The WHO’s *Guidance* posits that “simply living in the vicinity of a GMM release is not sufficient grounds to require informed consent from any individual for an open release of mosquitoes”. While the solicitation of individual informed consent in host settings of gene drive field trials may not be necessary, possible, or feasible in some instances, local community and stakeholder engagement will be key to building trust and facilitating the conduct of such research. In this regard, the approaches taken by investigators and sponsors of political science field research and weather modification field research should be avoided. Rather, proponents of gene drive field research should look to the *Eliminate Dengue* field trials, some cluster randomised trials, and some pragmatic clinical trials for guidance regarding how the solicitation of individual informed consent of host communities ought to be managed, and how research communities ought to be engaged. Trust-building and engagement initiatives should be undertaken by researchers and sponsors – preferably under the oversight of “an ethics advisory group comprising experts external to the project” [5] – not simply because engagement and trust are a means to an end, but because they constitute important ethical values in-and-of-themselves. Adopting such a mindset and taking such an approach could engender trust and facilitate understanding of this nascent field amongst communities, stakeholders, and publics.

## Data Availability

Publicly accessible sources.

## Abbreviations

CAPRISA: Centre for the AIDS Programme of Research in South Africa
Cas9: CRISPR-associated protein 9
CIOMS Guidelines: International Ethical Guidelines for Health-Related Research Involving Humans
CIOMS: Council for International Organizations of Medical Sciences
CRISPR: Clustered regularly interspaced short palindromic repeats
CRT: Cluster randomised trial
DOH: Declaration of Helsinki
GMM: Genetically modified mosquitos
Guidance: WHO guidance framework for testing of genetically modified mosquitoes
HIV/AIDS: Human immunodeficiency virus infection and acquired immune deficiency syndrome
JAS: Jerome Amir Singh
NASEM: US National Academy of Sciences, Engineering and Medicine
PCT: Pragmatic Clinical Trial
TB: Tuberculosis
UNESCO: United Nations Educational, Scientific and Cultural Organisation
US: United States of America
WHO: World Health Organisation
